# Transactions of the Medical Association of Georgia

**Published:** 1883-10

**Authors:** 


### TRANSACTIONS OF THE MEDICAL ASSOCIATION OF GEORGIA.

This publication is already before us. Dr. James A. Gray, the Secretary, deserves the hearty congratulations of the body and the profession for this unequaled promptness in the completion and delivery of the book.

The work was executed by James P. Harrison & Co., and for excellence of material, taste in binding and typographical beauty, and general accuracy, nothing of the kind could be more finished.

But while we admire this taste and style of the work, we can not but agree with Dr. Gaither, of Oxford, that a plainer and less pretentious book would do as well, and save no small sum to the too often restricted treasury of the Association.

In the September number of the Register we published, in advance, from the proof-sheets of the Transactions, two excellent papers from the respective pens of Drs. V. H. Taliaferro and G. H. Nobles, for which we now make the acknowledgement which was then inadvertantly omitted.

There are several other articles in the work of great merit, which we shall take pleasure in reproducing at the proper time.

The remaining contributions or reports are the Address of the President, R. P. Moore, M.D., of Forsyth; Report on Diseases of Women, from the First Congressional District, by R. J. Nunn, M.D., Savannah, Ga.; Uterine Diseases, Sometimes Called Consumption, by Ephraim Cutter, A.M., M.D., N. Y.; Report of Section on Surgery for the Seventh Congressional District, by I. C. Bivings, M.D., Dalton, Ga.; Climate, by A. Means, M.D., D.D., LL.D., Oxford, Ga.; Hyosciamin in the Treatment of Violent Mania, by T. O. Powell, M.D., Sup't. Ga. Lunatic Asylum; Hotz' Operation for Entropion and Trichiasis, with Fifteen Cases, by A. G. Hobbs, M.D., of Atlanta; Report of Surgical Cases, by Thos. R. Wright, M.D., of Augusta, Ga.; Acute Inflammations of the Throat, by Chas. W. Hickman, M.D., of Augusta, Ga.; Typhoid Fever, by L. G. Hardman, M.D., Harmony Grove, Ga.; Dysentery, by I. W. Duncan, M.D., Atlanta, Ga.

